# Fatty acids - from energy substrates to key regulators of cell survival, proliferation and effector function

**DOI:** 10.15698/cst2020.01.209

**Published:** 2019-12-10

**Authors:** Danilo Cucchi, Dolores Camacho-Muñoz, Michelangelo Certo, Valentina Pucino, Anna Nicolaou, Claudio Mauro

**Affiliations:** 1 Barts Cancer Institute, Queen Mary University of London, Charterhouse Square, London EC1M 6BQ, UK.; 2 Laboratory for Lipidomics and Lipid Biology, Division of Pharmacy and Optometry, Faculty of Biology, Medicine and Health, School of Health sciences, The University of Manchester, Manchester Academic Health Science Centre, Oxford Road, Manchester M13 9PT, UK.; 3 Institute of Inflammation and Ageing, College of Medical and Dental Sciences, University of Birmingham, Mindelsohn Way, Birmingham B15 2WB, UK.; 4 Lydia Becker Institute of Immunology and Inflammation, Faculty of Biology, Medicine and Health, The University of Manchester, Manchester Academic Health Science Centre, Oxford Road, Manchester M13 9PT, UK.; 5 Institute of Cardiovascular Sciences, College of Medical and Dental Sciences, University of Birmingham, Mindelsohn Way, Birmingham B15 2WB, UK.; 6 Institute of Metabolism and Systems Research, College of Medical and Dental Sciences, University of Birmingham, Mindelsohn Way, Birmingham B15 2WB, UK.

**Keywords:** fatty acids, immune cells, T cells, cancer cells, metastasis, cancer immunology

## Abstract

Recent advances in immunology and cancer research show that fatty acids, their metabolism and their sensing have a crucial role in the biology of many different cell types. Indeed, they are able to affect cellular behaviour with great implications for pathophysiology. Both the catabolic and anabolic pathways of fatty acids present us with a number of enzymes, receptors and agonists/antagonists that are potential therapeutic targets, some of which have already been successfully pursued. Fatty acids can affect the differentiation of immune cells, particularly T cells, as well as their activation and function, with important consequences for the balance between anti- and pro-inflammatory signals in immune diseases, such as rheumatoid arthritis, psoriasis, diabetes, obesity and cardiovascular conditions. In the context of cancer biology, fatty acids mainly provide substrates for energy production, which is of crucial importance to meet the energy demands of these highly proliferating cells. Fatty acids can also be involved in a broader transcriptional programme as they trigger signals necessary for tumorigenesis and can confer to cancer cells the ability to migrate and generate distant metastasis. For these reasons, the study of fatty acids represents a new research direction that can generate detailed insight and provide novel tools for the understanding of immune and cancer cell biology, and, more importantly, support the development of novel, efficient and fine-tuned clinical interventions. Here, we review the recent literature focusing on the involvement of fatty acids in the biology of immune cells, with emphasis on T cells, and cancer cells, from sensing and binding, to metabolism and downstream effects in cell signalling.

## INTRODUCTION

Lipids are important biomolecules involved in a plethora of biological processes, spanning from the production and storage of energy [1] and assembly and function of the cellular membranes [2] to activation of genes [3] and modulation of signalling pathways [4]. This diverse group of compounds comprises fatty acids, glycerolipids, glycerophospholipids, sphingolipids, sterol lipids, prenol lipids, saccharolipids and polyketides [5–7]; typically, the cellular lipidome comprises more than 2,000 species. This structural diversity endows lipids with varied properties that enable and support a plethora of structural and functional roles.

As lipids are not encoded, their levels are regulated both by nutritional intake and biosynthetic pathways found in almost all cell types. Fatty acids are simple lipids biosynthesised by the complex enzyme fatty acid synthase (FAS) through the sequential elongation of acetyl-CoA [8]. The resulting 16-carbon (C) acyl chain palmitic acid can be further elongated and desaturated. However, humans and other animals require nutritional intake of two essential fatty acids -linoleic acid and alpha-linolenic acid - precursors to long and very long polyunsaturated fatty acids (PUFA). As well as contributing to the structural diversity of membrane glycerophospholipids and sphingolipids, PUFA are metabolised to potent autacoids, hormone-like mediators known for their involvement in inflammation and immunity [9–11], while the catabolism of fatty acids via β-oxidation provides the cells with an efficient way for energy production. Membrane glycerophospholipids and sphingolipids, as well as acylglycerols (e.g. triacylglycerols) act as cellular pools of esterified fatty acids that can be liberated and further metabolised to meet the energy and other biosynthetic needs of different cell types.

In this review, we are discussing the involvement of fatty acids in immunity, particularly in T cells, and cancer, with focus on their roles as energy substrates, regulators of cell survival, proliferation and effector function. Increasing evidence supports the notion that fatty acids can influence the biological behaviour of immune and other cell types when involved in pathophysiological conditions, such as metabolic disorders, autoimmune diseases and cancer; therefore, exploring the metabolism and properties of fatty acids can provide new potential pharmacological targets in the treatment of different clinical conditions.

## FATTY ACIDS IN IMMUNITY

**Figure 1:**
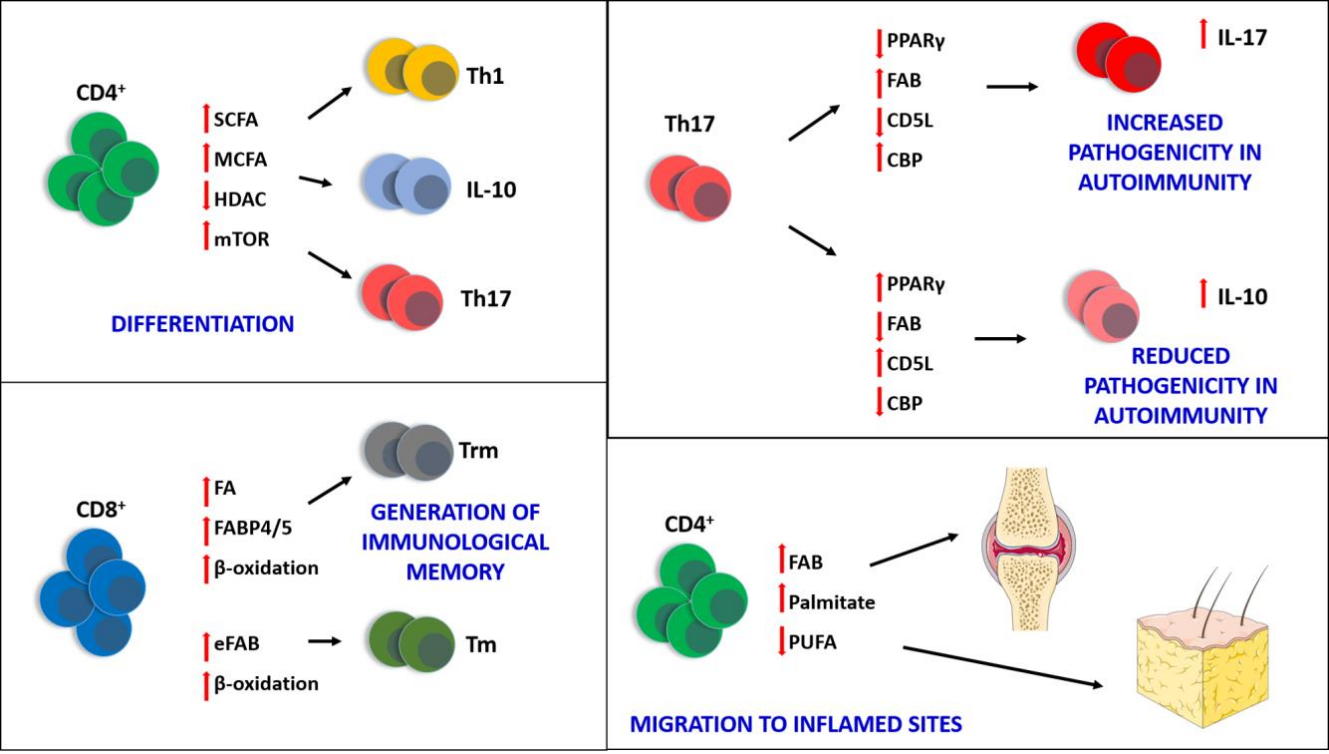
FIGURE 1: Fatty acids are crucial to many biological processes occurring in immune cells, particularly T lymphocytes. Short (C2-4)- and medium (C6-14) chain fatty acids (SCFA and MCFA) are able to influence the differentiation of CD4^+^ in Th1, Th17 and IL-10^+^ T cells, by downregulating histone deacetylase (HDAC) activity and activating mammalian target of rapamycin (mTOR). The generation of CD8^+^ tissue resident memory T cells (T_rm_) also relies upon the availability of fatty acids (FA) available for β-oxidation and the presence of the fatty acid binding proteins FABP4 and FABP5. The generation of central memory CD8^+^ T cells (T_m_) instead relies upon endogenous fatty acid biosynthesis (eFAB), which also provides substrates for β-oxidation, crucial for the long-term survival of these cells. The pathogenicity of Th17 can be also regulated by fatty acids: by increasing the activity of fatty acid biosynthesis (FAB) and cholesterol biosynthesis (CBP), and downregulating the expression of PPARγ and CD5L, Th17 cells produce IL17 and become more pathogenic, especially in autoimmune diseases such as multiple sclerosis and rheumatoid arthritis. Conversely, upregulation of PPARγ and CD5L, and blocking of FAB and CBP, can reduce the pathogenicity of Th17 and increase production of IL10. Finally, saturated fatty acids (e.g. palmitate) can enable the migration of T cells towards non-lymphoid inflamed sites (e.g. joints in arthritis and fat tissue in obesity) where they sustain chronic inflammation; polyunsaturated fatty acids (PUFA) can have the opposite effect and inhibit T cell migration, exerting anti-inflammatory properties.

Growing evidence points to the importance of lipid metabolism and signalling in the function of different types of immune cells, both in a homeostatic state and during the immune response. Fatty acids and their derivatives are of particular importance and recent discoveries have highlighted their role in the regulation and function of immune cells, in particular T lymphocytes **(Figure 1)**.

### Sensing and binding

Fatty acids bind to several surface receptor members of the family of G protein coupled receptors (GPCR) [12, 13]. Of particular interest is GPR84, which has affinity for fatty acyl chain length of 6-14 C (medium chain fatty acids) and is expressed by a variety of immune cells, mainly neutrophils and monocytes/macrophages, and to a lesser extent, by CD4^+^ and CD8^+^ T cells. Expression of GPR84 both in human and mouse monocytes is upregulated upon lipopolysaccharide (LPS) stimulation, suggesting that signals triggered by medium chain fatty acids may have a role in monocyte/macrophage activation [14]. Moreover, medium chain saturated fatty acids such as caproic acid (C6), undecanoic acid (C11) and lauric acid (C12), are able to stimulate the secretion of interleukin 12 p40 subunit (IL12 p40) in mouse LPS-stimulated RAW264.7 cells, showing that they can have a role in regulating the activity of different immune cells during inflammation [14].

It has been recently reported that the short chain fatty acids acetate (C2), propionate (C3) and butyrate (C4) are able to orchestrate the differentiation of CD4^+^ T cells in effector or regulatory cells modulating the activity of histone deacetylases (HDAC) [15]. Short chain fatty acids can also inhibit HDAC in T cells leading to the amplification of the mTOR pathway, through acetylation of p70 S6 kinase and consequent phosphorylation of rS6, all events required for the differentiation in T helper 17 (Th17), T helper 1 (Th1) and IL-10^+^ T cells [15]. As short chain fatty acids can be readily uptaken through the plasma membrane [16], their effect was found to be independent of the surface receptors GPR41 and GPR43, which can sense acetate, butyrate and propionate [17], and have been shown to be important in regulating intestinal inflammatory responses [18]. These findings identify short chain fatty acids as crucial gut metabolites affecting the balance of effector and regulatory T cells and orchestrating the immune response in the gut.

Long chain fatty acids (14-22 C), such as the saturated palmitic acid (C16:0) and monounsaturated oleic acid (C18:1), can also be sensed by CD36, a fatty acid translocase required for the uptake of fatty acids in the gut [19], liver [20], myocardium, skeletal muscle and adipose tissue [21, 22]. CD36 expression in endothelial cells (EC) is instrumental for the optimal translocation of long chain fatty acids from circulation to cardiomyocytes, skeletal muscle and adipose tissue, with important consequences for fatty acid metabolism, glucose utilization, glucose tolerance and insulin sensitivity [23]. CD36 also binds to a range of lipidic ligands, such as oxidized phospholipids [24], oxidized LDL [25] and native lipoproteins [26]. CD36 was found to be particularly important for the activity of phagocytes during inflammation, by functioning as a scavenger receptor [27] and by cooperating with several Toll-like receptors [28–30]. CD36 mutations in humans have been associated with CD36 deficiency in platelets and monocytes, with potential consequences for their functions [31]. CD36 loss has been reported to reduce macrophage infiltration in adipose tissue [32], while pharmacological inhibition of CD36 *in vitro* reduces saturated fatty acid uptake (e.g. palmitic acid (16:0) and stearic acid (18:0)) in macrophages and ameliorates insulin signalling in adipocytes. More importantly, genetic ablation of CD36 in the hematopoietic compartment led to a reduced infiltration of macrophages and improved insulin signalling in the adipose tissue of mice fed a high fat diet (HFD) [32], although it did not reduce the accumulation of long chain fatty acids [32, 33], suggesting that some of the CD36-mediated functions in macrophages do not depend on its fatty acid translocase activity. All these findings highlight the importance of CD36 as a target for the treatment of metabolic disorders with an inflammatory component, such as obesity and diabetes. T cells also express CD36 on their surface, with T memory (T_m_) cells showing lower levels than T effector (T_eff_) cells [34].

Fatty acid binding proteins (FABP) are a family of intracellular and extracellular proteins that bind saturated and unsaturated fatty acids [35]. It is now clear that these proteins not only buffer and transport fatty acids, but are also deeply involved in the regulation of their metabolism with consequences for cell signalling, particularly during inflammation [36, 37]. Recently, tissue-resident memory T_rm_ cells have been shown to be dependent on the activity of FABP4 and FABP5 for long-term survival. Pan *et al.* [38] demonstrated that the deficiency of FABP4/5 impairs the uptake of fatty acids such as palmitate, by skin CD8^+^ T_rm_ cells, thus reducing their long-term survival *in vivo*. CD8^+^ T_rm_ cells lacking FABP4/5 fail to increase mitochondrial oxidative metabolism in the presence of fatty acids *in vitro*, and their persistence *in vivo* was significantly reduced due to inhibition of β-oxidation. Finally, FABP4 and FABP5 were also found upregulated in human CD8^+^ T_rm_ cells isolated from normal and psoriatic skin, confirming the importance of fatty acids in the maintenance and longevity of this tissue-resident protective immune population [38].

Cellular fatty acids and their metabolites activate different signals via binding peroxisome proliferator-activated receptors (PPAR), nuclear receptors involved in the regulation of transcription of genes linked to lipid metabolism [39]. PPARα and β/δ are particularly important in cardiac muscle, brown adipose tissue and liver, whilst PPARγ is more ubiquitously expressed [40–42]. These receptors have been proven to be important in the differentiation of a number of T cell subsets [43], particularly in informing the decision of CD4^+^ T cells toward differentiating to Th17 or T regulatory (T_reg_) cells [44]. Consistently, Klotz *et al.* [45] have shown that PPARγ regulates the differentiation of Th17 T cells, by negatively controlling the activity of RORγt. The same report shows that loss of PPARγ increases the severity of experimental autoimmune encephalomyelitis (EAE) and multiple sclerosis in mouse models, leading to a greater infiltration of Th17 cells into the central nervous system [45]. Overall, these findings indicate that activation of PPARγ with selective agonists can inhibit the differentiation of Th17 cells in autoimmune conditions with a strong Th17 component, such as multiple sclerosis, but also rheumatoid arthritis and psoriasis, making PPAR receptors a very promising pharmacological target in autoimmunity.

PPARγ was also found to be crucially important for the activity of adipose tissue associated- T_reg_ cells, which express PPARγ at higher level than T_reg_ originating from lymphoid organs [46]. Expression of PPARγ was associated with a cluster of mRNAs involved mainly in leukocytes migration and extravasation (*Ccr1*, *Ccr3*, *Cxcr6*, *Cxcl2* and *Cxcl3*), lipid metabolism (*Pcyt1a*, *Dgat1*) and *Il10* transcript. Expression of Foxp3 and PPARγ in naïve CD4^+^ T cells was sufficient to induce the same cluster of mRNAs while additional treatment with PPARγ agonist pioglitazone and rosiglitazone further enhanced the enrichment of transcripts involved in fatty acid transport (*Cd36*, *Slc27a2*), biosynthesis (*Lipe*, *Scd1*) and oxidation (*Cpt1a*). Genetic deletion of PPARγ *in vivo* led to a contraction of the T_reg_ population in adipose tissue with a relative increase in the number of pro-inflammatory macrophages. Furthermore, in obese mice, treatment with pioglitazone enhanced the accumulation of T_reg_ in epididymal adipose tissue and their expression of CD36. This phenotype was abrogated in obese PPAR deficient mice, which also showed a less marked reduction of pro-inflammatory macrophages upon treatment with pioglitazone, as compared to their wild type (wt) counterpart. PPARγ deficient mice did not show an improvement in metabolic parameters (insulin resistance, glucose and insulin tolerance) when treated with pioglitazone [46]. These findings clearly demonstrate that PPARγ is a crucial regulator of the properties of adipose tissue associated- T_reg_ cells and its expression in T_reg_ is necessary for the insulin-sensitizing activity of pioglitazone, with important consequences for the management of obesity-induced insulin resistance.

### Activation and differentiation

T lymphocytes undergo a clonal expansion upon antigen recognition, which is necessary to mount an appropriate immune response. This process demands not just energy, but also the activation of specific signals required for the proper differentiation and activation of the cell. It has been shown that activation of the T cell receptor (TCR) is accompanied by upregulation of genes involved in the biosynthesis of cholesterol and fatty acids [47]. This anabolic programme is orchestrated by sterol regulatory element binding protein (SREBP) [47]. Interestingly, lack of SREBP by genetic inactivation is particularly detrimental to T cells undergoing clonal expansion after activation, as it does not allow for biosynthesis of cholesterol and fatty acids required to sustain their energy demands. Moreover, SREBP is required for the biosynthesis of cellular membranes, as shown by experiments where addition of cholesterol was able to rescue the growth of T cells [47]. Also, *in vivo*, the lack of SREBP was responsible for a poor anti-viral response against the lymphocytic choriomeningitis virus, again demonstrating that the ability of activating an anabolic lipid programme for the biosynthesis of cholesterol and fatty acids is a requirement for a proper immune response [47].

The generation of T_m_ cells has also been shown to rely upon mitochondrial fatty acid β-oxidation [34]. It has been shown that T_m_ cells do not take up extracellular palmitate, unlike T_eff_, highlighting how different T cell subtypes express different preferences for fatty acid substrates. Survival of T_m_ cells was reduced in the presence of the FAS inhibitor C75 [48], confirming that these cells rely upon *de novo* fatty acid biosynthesis for survival. Moreover, T_m_ cells utilize glucose to newly synthesise fatty acids to fuel oxidative phosphorylation, thus being independent from extracellular fatty acid uptake. The lipolysis occurring in these cells is crucial to their function, and it has been demonstrated that in T_m_ cells, lipids associate with lysosomes, where lysosomal acid lipase (LAL) produces fatty acids necessary to support β-oxidation. Furthermore, loss of LAL activity reduced the survival of T_m_ cells, without affecting T_eff_ cells, and also inhibited the development of T_m_ cells upon infection *in vivo* [34].

Michalek et al. [49] have reported that T_eff_ cells and T_reg_ cells exhibit different metabolic requirements for differentiation and function, with the former being more glycolytic and the latter relying more on lipid oxidation [49], while Berod *et al.* [50] showed how *de novo* fatty acid biosynthesis plays a crucial role in determining the differentiation of CD4^+^ T cells to Th17 or T_reg_ Foxp3^+^ cells. These authors showed that inhibition of acetyl-CoA carboxylase (ACC) *in vitro*, using the specific inhibitor Sorafen A, leads to an impaired differentiation of Th17, favouring instead the differentiation of Foxp3^+^ T_reg_ cells. CD4^+^ naïve T cells, cultured in Th17 polarizing conditions in the presence of Sorafen A, failed to increase the production of IL-17 and upregulate genes associated with this lineage, such as *Hif1alpha* and *Stat3*. To further evaluate the role of ACC as a potential therapeutic target, the phenotype of mice lacking ACC1 (cytosolic isoform of the carboxylase required for the *de novo* synthesis of fatty acids [51]) in T cells was analysed. When compared to wt mice, and after induction of EAE, mice lacking ACC1 were found to be protected and did not develop any clinical signs of the disease. Similarly, administration of the ACC1 inhibitor Sor-S1036 in wt mice affected by EAE, resulted in a significant delay in disease onset and severity [50].

The possibility of exploiting ACC1 as a promising pharmacological target has also been shown in the context of chronic malaria infection [52]. Injection of CD4^+^ T cells lacking ACC1 in mice infected with *Plasmodium chabaudi*, resulted in reduced cell survival and was accompanied by reduced generation of T_m_ cells. A similar result was obtained using TOFA, an inhibitor of ACC1, during T cell priming that increased the number of T_eff_ cells and reduced parasitemia. Inhibition of fatty acid synthesis was shown to be particularly detrimental for T_m_ cells as they rely more on newly synthesised fatty acids as compared to T_eff_ cells, suggesting that targeting fatty acid biosynthesis in T_m_ cells may be important in the development of new strategies for fighting chronic infections like malaria [52]. These findings highlight the importance of fatty acid metabolism for the generation of immunological memory and suggest that regulation of this pathway is an interesting prospect for the development of vaccines.

The importance of fatty acids in balancing the protective effects of T cell responses and their pathogenicity was addressed by Wang *et al.*, with particular regard to Th17 cells, which can lead to autoimmunity when they are aberrantly activated [53]. In this study, CD5L, a member of the scavenger receptor cysteine-rich superfamily [54], was identified in a single cell RNA-seq screening as a regulator of pathogenicity in Th17 cells and was found expressed only in non-pathogenic Th17 cells, both *in vitro* and *in vivo.* CD5L^-/-^ T cells injected in mice affected by EAE led to a more severe phenotype, whilst mice that received wt cells did not show any clinical signs of disease. As CD5L can bind to FAS [55], the lipidome of wt and CD5L^-/-^ Th17 cells was analysed and showed two completely distinct fatty acid profiles: the presence of CD5L increased the levels of PUFA versus saturated fatty acids and mono-unsaturated fatty acids, and reduced cholesterol content. In detail, CD5L is able to suppress the cholesterol synthesis pathway. These changes affect the activity of RORγt by modulating the availability of its ligands and affecting its ability to bind to target genes, such as *IL-17a* and *IL-23r*, whilst increasing binding to *IL-10*, making of CD5L a repressor of pathogenicity of Th17 cells [53]. Overall, these findings show how potent the effect of fatty acid metabolism is on the biology of immune cells, not only as source of energy, but also by sustaining the molecular signals required for differentiation and function.

Direct exposure to palmitate was found to be responsible for the biased differentiation of CD4^+^ T cells towards the expansion of a detrimental, pro-inflammatory effector memory population in obesity [56]. These aberrant T lymphocytes eagerly migrate to non-lymphoid, inflammatory sites (such as the fat tissue during obesity), where they sustain a low-grade chronic inflammation which is a hallmark of many metabolic disorders, such as obesity, type II diabetes and atherosclerosis [56].

In obesity, there are many different immune cells that play a role in sustaining the inflammatory response within the adipose tissue. The stress response triggered by hypertrophy in both adipocytes and stromal cells [57, 58] is responsible for the recruitment and activation of CD8^+^ T cells, Th1 cells, and natural killer (NK) cells, leading to the accumulation of pro-inflammatory M1 macrophages [59–61]. In 2016, another population of immune cells was demonstrated to play an important role in the maintenance of obesity, the group 1 innate lymphoid cells (ILC1) [62]. The authors showed that residing ILC1 proliferate and accumulate locally, in the adipose depots, during HFD-induced obesity and are the main producer of IFN-γ. The sustained production of IFN-γ is responsible for the polarization of macrophages towards a pro-inflammatory M1 phenotype which then contributes to the development of obesity-associated insulin resistance. The axis IL12-STAT4 was found to be the molecular switch for the production of IFN-γ and the consequent M1 polarization of macrophages [62]. This is another example of how fat accumulation can deeply change the biological behaviour of immune cells, interfering with their homeostatic function, with great consequences for the establishment of pathological conditions.

B lymphocytes have also been shown to have an important role in the development of age-induced fat accumulation and insulin resistance [63]. Carter and co-workers [63] found that aging mice (between 6 and 12 months old) have higher number of follicular B2 cells in circulation and epididymal white adipose tissue. The expansion of this B cell population correlated with a higher plasma level of IgG (in particular IgG2c) and glucose intolerance. Moreover, these cells displayed a greater expression of OcaB (B-cell-specific nuclear cofactor Oct coactivator – crucial for B cell maturation [64] and IgG production [65]). Genetic ablation of OcaB resulted in the abrogation of this phenotype, with improvement of glucose intolerance and insulin sensitivity, and reduced fat accumulation during aging due to increased energy expenditure. OcaB^-/-^ mice showed a higher core body temperature and increased metabolic activity in their adipose tissue depots, as shown by enhanced uptake of bromopalmitate and glucose. Replenishment of OcaB^-/-^ mice with OcaB^+/+^ B cells, via bone marrow transfer, was sufficient to re-induce body weight gain, glucose intolerance and insulin insensitivity [63].

Recently, omega-3 (n-3) PUFA have been shown to alter the trafficking of activated CD4^+^ T cells to fat tissue [66]. Eicosapentaenoic acid (EPA) and docosahexaenoic acid (DHA) were both able to reduce the number of effector memory CD4^+^ T cells and change the array of bioactive lipids produced in lymphoid organs and adipose tissues of animals following nutritional supplementation. EPA and DHA were also able to prevent the polarisation of T cells and activation of the small Rho GTPases Rhoα and Rac1, both events required for migration to target/inflamed tissue [66]. EPA and DHA have also been shown to increase the infiltration of CD4^+^ and CD8^+^ T cells in UV irradiated human skin, potentially through changes in the network of cutaneous bioactive lipids [67].

N-3 PUFA have also been shown to boost B cell antibody production [68] and B cell response [69], but the differential contribution of EPA and DHA to this phenotype was only recently addressed by Teague *et al.* [70]. The authors showed that a diet enriched with either EPA or DHA resulted in accumulation of EPA and DHA ethyl esters in B cells at the expense of n-6 PUFA. EPA and DHA were also able to increase the frequency of specific subsets of B cells (in particular IgM^+^ IgD^-^ CD21^low^ CD23^-^ B cells) and while both of them increased the production of IgM, only EPA boosted the production of IgA. Moreover, after ten weeks supplementation, EPA and DHA increased B cell TNFα and IL6 production, but only DHA induced the production of IL10 [70]. The differential activity of n-3 PUFAs on B cells bears important consequences for application in clinical settings, being now clear that EPA and DHA are not biologically equivalent.

The availability of fatty acids can impact the function of many proteins through post translational modifications including S-acylation [71]. The most common protein acylation is palmitoylation although use of different acyl groups has been reported [72, 73]. Palmitoylation is catalysed by aspartate–histidine–histidine–cysteine (DHHC) acyl transferases and can deeply affect the trafficking, localisation and activity of proteins [74]. Recently, Chopard *et al.* have highlighted how palmitoylation is important for HIV-1 infection [75]. The viral protein Tat can be secreted by infected cells and subsequently be endocytosed by many different cell types altering the expression of genes related to HIV-associated cancers [76]. Tat can also inhibit the cellular processes that rely upon phosphatidylinositol (4,5) bisphosphate (PI(4,5)P2) for phagocytosis and neurosecretion [77]. Accumulation of Tat in uninfected cells (e.g. T cells, macrophages and neurosecretory cells) is attributed to its palmitoylation by S-acyl transferase DHHC-20 on Cys31, which stabilises the interaction with PI(4,5)P2 thus preventing Tat secretion. The persistence of Tat on plasma membranes and its stable interaction with PI(4,5)P2 interferes with PI(4,5)P2-dependent membrane trafficking, with important consequences for Tat-mediated toxicity in HIV-1 infected individuals [75].

## FATTY ACIDS IN CANCER

It is widely accepted that cancer cells undergo a profound metabolic reprogramming, which is now considered a hallmark of the disease [78]. The so-called Warburg effect is one of the most remarkable metabolic phenotypes of cancer cells, consisting of prompt uptake of glucose and upregulation of glycolysis even in the presence of oxygen, with consequent production of lactate and its release into the tumour microenvironment [79, 80].

**Figure 2:**
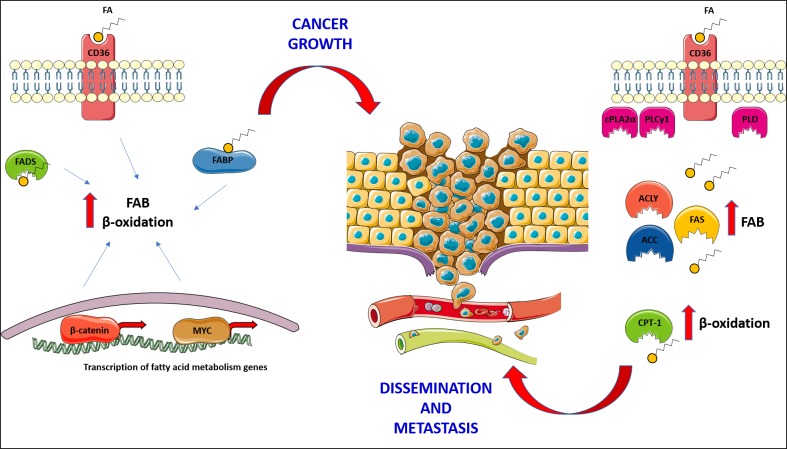
FIGURE 2: Cancer cells rely upon fatty acids (FA) for proliferation, survival and metastasis, and utilise them for the production of energy and membrane maintenance; consequently, both fatty acid biosynthesis (FAB) and β-oxidation are found increased in many cancers. The expression of CD36 and fatty acid binding proteins (FABP) has been associated with cancer growth. The activity of fatty acyl-CoA desaturases (FADS) sustains FAB, promoting cancer growth and survival. As shown on the left side of the figure, some cancers put in place a transcriptional programme, governed by master regulators such as MYC and β-catenin, in order to boost the activity of FAB and β-oxidation and, generally, utilisation of fatty acids, supporting cancer growth. Finally, on the right side of the figure how cancer cells utilise fatty acids to support their migration and growth in secondary sites is shown; in this context, the expression of CD36 is central for the dissemination of cancer cells from the primary tumour and the establishment and growth of metastasis. The activity of enzymes involved in phospholipid hydrolysis (Phospholipase D – PLD; cytosolic phospholipase A2α – cPLA2α; phospholipase Cγ1 – PLCγ1), in fatty acid biosynthesis (ATP citrate lyase – ACLY; acetyl-CoA carboxylase – ACC; fatty acid synthase – FAS) and in their catabolism (Carnitine palmitoyl transferase 1 – CPT-1), has been proven fundamental for dissemination and metastasis establishment.

Growing evidence demonstrates the importance of lipid metabolism and lipid signalling in cancer cell biology, highlighting the great potential of lipid metabolising enzymes, transporters and receptors as therapeutic targets. Cancer cells show changes in fatty acid and cholesterol metabolism, and this impacts upon their ability to grow and proliferate [81–83] **(Figure 2)**.

### Sensing and binding

Fatty acid sensing is crucial for cancer cells, and different studies have shown the importance of lipoprotein receptors and FABP for cancer cell survival [84–87]. Saturated and unsaturated long chain fatty acids differentially regulate the transcriptional activity of the retinoic acid receptor RAR and PPARβ/δ, through FABP5. Although saturated fatty acids block FABP5 and inhibit PPARβ/δ, unsaturated fatty acids activate PPARβ/δ. The result is that saturated fatty acids, by activating RAR and inhibiting PPARβ/δ, are able to suppress the growth of cancer cells [88]. Recently, a role for FABP5 in promoting cancer growth through the estrogen-related receptor α (ERRα)-dependent upregulation of metabolic genes has been reported in prostate cancer [89].

CD36 represents another promising pharmacological target for interfering with the fatty acid uptake by cancer cells, and very recently, Watt and co-workers showed how targeting CD36 could be an effective strategy against prostate cancer [90]. In this study, the authors used patients' samples and xenograft models to demonstrate that increase in uptake and utilization of fatty acids in prostate cancer is due, at least in part, to expression of CD36, and that the presence of this transporter correlates with aggressive disease. Deleting CD36 or using a blocking monoclonal antibody slowed cancer progression and disease severity [90].

### Biosynthesis and metabolism

Fatty acid biosynthesis plays a pivotal role in cancer biology and it is not surprising that inhibition of this process represents an appealing pharmacological avenue [91]. SREBPs have been shown to be important for cancer survival and progression [92, 93]. More recently, Li *et al.* [94] have pointed to their importance in hepatocellular carcinoma (HCC). By blocking SREBP, either genetically or pharmacologically, the progression of HCC was remarkably inhibited together with downregulation of tumour-promoting inflammatory cytokines, such as IL-6, TNFα and IL-1β [94].

Inhibition of FAS, leading to impaired *de novo* biosynthesis has been explored as a therapeutic strategy for metastatic castration-resistant prostate cancer (mCRPC) [95], as demonstrated by the development of inhibitors that reduced the growth of human CRPC xenograft and human mCRPC-derived organoids [95]. The importance of fatty acid biosynthesis was further addressed in a recent study showing how cancer cells can utilize different fatty acyl-CoA desaturases (FADS) in order to sustain proliferation [96]. Several cell lines, including mouse HCC and primary human liver and lung cancer cell lines, were found to be independent from the activity of stearoyl-CoA desaturase (SCD), instead they were able to desaturate palmitate to the unusual sapienic acid (rather than to palmitoleic acid) through the activity of FADS2 [97]. Inhibition of both desaturases, SCD and FADS2, resulted in reduced cancer growth *in vivo*, suggesting that targeting of multiple pathways may be needed in order to effectively impair cancer metabolic plasticity [96].

Utilising fatty acids for energy production, in particular via β-oxidation, is important for cancer cell growth and has been considered a target for the development of new therapies in various cancers including B cell lymphoma [98], leukemia [99], prostate cancer [100], ovarian cancer [101] and pancreatic cancer [102]. Of particular interest in this context is the understanding of the transcriptional regulation of β-oxidation in cancer, as this has been associated with the over-expression of certain oncogenic transcription factors, such as MYC and β-catenin.

The oncogene MYC is responsible for alteration of metabolism during tumorigenesis [103, 104]. Recently, Camarda *et al.* [105] showed that MYC-overexpressing triple-negative breast cancer (TNBC) is highly dependent on β-oxidation for survival. To assess the role of MYC in TNBC, the authors analysed the metabolites produced by breast tumours and normal mammary gland from a conditional transgenic model of MYC-overexpressing tumours and reported that MYC-TNBC tumours are enriched in acylcarnitines, metabolites of the β-oxidation pathway [105]. In human samples, TNBC tumours also appear to upregulate genes responsible for the activation of β-oxidation and downregulate genes of fatty acid biosynthesis, both at mRNA and protein level. Finally, inhibiting β-oxidation, either using the inhibitor etomoxir or knocking-down carnitine palmitoyl transferase 1B, proved to be detrimental for the energy production and survival of cancer cells *in vitro* and also *in vivo* [105].

Of particular interest is also the finding of β-catenin (CTNNB1)-mutated HCC dependence on fatty acids [106]. It was reported that these tumours do not comply with the Warburg effect, they are not glycolytic, and activate a strong fatty acid catabolic programme by increasing the flux of β-oxidation. This phenotype was observed in mice recapitulating β-catenin mutated HCC (by inactivation of Apc - [107]) and further confirmed in human samples, where the same metabolic signature was found. Loss of PPARα, a downstream target of β-catenin, was sufficient to blunt the upregulation of β-oxidation and reduce the formation and progression of tumours. Pharmacological inhibition of β-oxidation with etomoxir exerted the same effect on tumour growth [106].

### Cancer immunology

The importance of fatty acid metabolism in regulating the functions of immune cells was recently shown to influence the ability of the immune system to clear tumours. The interaction between immune and cancer cells takes place in the tumour microenvironment, which is able to influence the proliferation and activation of immune cells. Pacella *et al.* have shown how the tumour microenvironment favours the expansion of a T_reg_ cell population over a conventional T cell (T_conv_) population, which would be able to fight the tumour [108]. T_reg_ are able to survive better than T_conv_ in the tumour microenvironment because of their ability to utilize glucose for fatty acid biosynthesis. T_reg_ indeed rely on fatty acid biosynthesis more than their conventional counterparts and the authors confirmed that activated T_reg_ isolated from human liver cancers express a unique gene signature supporting glycolysis and lipid biosynthesis [108].

The relationship between lipid metabolism, cancer and the immune system has become the focus of growing research activity in recent years, following epidemiological studies revealing the association of cancer and obesity, and showing that obesity is a potent risk factor for developing cancer [109, 110]. Recently, a new mechanism explaining how fatty acid accumulation blunts the immune response in obesity has been described, suggesting that obesity can significantly impair the cytotoxic activity of NK cells [111]. Feeding mice a HFD altered in NK cells the expression of genes involved in lipid metabolism and trafficking, and upregulated PPARβ/δ target genes involved in lipid-droplet formation (*Lipe* and *Plin2*) and lipid uptake (*Cd36*, *Lpl* and *Lrp4*). Human NK cells from obese patients were found to accumulate more lipid droplets and were less able to kill cancer cells, an effect mediated via lipid driven mTORC1 inhibition. Blocking the translocation of fatty acids to mitochondria using etomoxir was sufficient to restore the cytotoxic activity of NK cells [111].

However, obesity is also well known to induce a state of low-grade chronic inflammation, with prolonged and exacerbated innate and adaptive immune responses [56, 112], which may promote obesity-driven diseases, including cancer. How these aspects of obesity control of innate and adaptive response are reconciled, and whether the effect on NK is specific to this cell type, are points of interest and remain unanswered.

Palmitoylation was found to be important in the context of cancer immunotherapy. Yao and colleagues demonstrated that programmed death ligand 1 (PD-L1), one of the main targets in cancer immunotherapy [113], can be palmitoylated in colorectal cancer cells by DHHC3 and that this modification increases its stability by preventing lysosomal degradation [114]. Inhibition of PD-L1 palmitoylation, either pharmacologically or by genetic ablation of DHHC3, was able to increase the T cell cytotoxicity against cancer cells *in vitro*, and to reduce tumour growth also *in vivo*. The authors developed a synthetic peptide able to specifically target DHHC3 and block PD-L1 palmitoylation, demonstrating that targeting this post-translational modification can be a feasible way of suppressing PD-L1-dependent immune evasion of tumour cells [114].

### Metastatic disease

Of particular relevance, especially from a clinical and therapeutic standpoint, is the association between dysregulated fatty acid metabolism and cancer metastasis. Several studies have highlighted the importance of fatty acids in the growth of secondary tumours, showing how enzymes involved in fatty acid biosynthesis and catabolism are crucial in this process **(Figure 2)**. In the context of fatty acid synthesis, the enzyme ATP citrate lyase (ACLY – responsible for the conversion of citrate to oxaloacetate and cytosolic acetyl-CoA) has been associated with metastatic disease in gastric adenocarcinoma (GA) [115]. It has been proposed that ACLY could be used as biomarker for the prediction of progression and prognosis of GA, as it was found overexpressed in patients' cancer tissue as compared to normal tissues, and its expression correlated negatively with patient survival [115]. In another study, ACLY was shown to be a potential therapeutic target. Indeed, its inhibition by microRNA-22 was able to suppress growth and invasion in different types of cancer cells [116]. In clinical samples, the authors observed that upregulation of ACLY correlates with the downregulation of miR-22. Moreover, in animal models of osteosarcoma and prostate cancer, treatment with miR-22 reduced tumour growth and formation of distant metastasis, and prolonged survival [116]. ACLY was also found to be a novel interactor of the low molecular weight isoform of cyclin E (LMW-E). This interaction allows breast cancer cells to store lipids necessary for growth, migration and invasion both *in vitro* and *in vivo*, while in clinical samples a strong correlation between worse prognosis, expression of LMW-E and accumulation of lipids droplets (a consequence of the increased activity of ACLY) was observed [117].

Another enzyme that could prove to be a good diagnostic marker as well as a potential therapeutic target is the ACC (responsible for the carboxylation of acetyl-CoA to malonyl-CoA). Expression of phospho-ACC was found to correlate with a worse overall survival in squamous cell carcinoma of the head and neck, in patients with node metastasis [118]. Recently, ACC was associated with aggressiveness in HCC and also with poor survival and disease recurrence, making it a potential prognostic marker and therapeutic target as well [119].

FAS is upregulated and associated with malignant progression in cancer: inhibition of FAS reduced invasion and migration of HCC [120], and was found responsible of promoting peritoneal metastasis in ovarian cancer through induction of epithelial to mesenchymal transition (EMT) [121]. Similarly, breast cancer cells undergo EMT in a FAS -dependent manner [122]. FAS was also associated with Wnt signalling and metastatic progression in colorectal cancer [123]. In the context of prostate cancer, Ahmad and co-workers found that PPARγ-mediated expression of FAS sustains the growth of prostate cancer cells and confers poor prognosis for metastatic prostate cancer [124]. Furthermore, Sounni *et al.* have shown how inhibition of FAS can confer advantage in overcoming tumour adaptation to anti-angiogenic treatment [125]. In this study, the authors found that interrupting the administration of anti-angiogenic drugs, such as sunitinib and sorafenib, led to a shift in the tumour metabolism responsible for an increase in fatty acid biosynthesis associated with tumour re-growth and dissemination, which often occurs in cancer patients after anti-angiogenic treatment withdrawal [125]. Pharmacological or genetic inhibition of FAS was able to suppress tumour growth and dissemination in different cancer models, highlighting a potential new strategy for treating cancer after withdrawal of anti-angiogenic drugs.

Phospholipid hydrolysis is important for metastatic progression, thus providing different potential targets for therapeutic intervention. Phospholipase D (PLD), through the release of phosphatidic acid and cooperation with Grb2 and Rac2 (events that affect the plasma-membrane plasticity and actin polymerization), was found to play a role in metastasis in several cancers. Henkels *et al.* [126] have demonstrated that PLD drives tumour growth and metastasis formation in a human breast cancer xenograft model. Deletion of PLD2 from metastatic cells suppressed the formation of metastasis and, consistently, its overexpression in non-metastatic cells led to the acquisition of a malignant metastatic phenotype. Furthermore, pharmacological inhibition of PLD2 was sufficient to suppress tumour growth and dissemination [126] while PLD2 has also been shown to regulate the expression of HIF1α in renal cancer cells [127]. Other phospholipases have been found to be important in metastasis formation; pharmacological inhibition of cytosolic phospholipase A2α (cPLA2α) with the inhibitor CIX, was able to reduce the migratory capabilities of a murine metastatic breast cancer cell line by interfering with Toll-like receptor- and type I interferon-mediated signals [128]. cPLA2α was found overexpressed also in human breast cancer samples and in invasive breast cancer cell lines [129]. Its inhibition significantly reduced the migration of metastatic cell lines *in vitro*, whilst its stable knockdown inhibited the EMT necessary for the acquisition of the migratory phenotype. Indeed, cPLA2α was found to be necessary for the TGFβ-induced EMT by activating the PI3K/Akt/GSK3β pathway, both *in vitro* and *in vivo* [129]. Phospholipase Cγ1 (PLCγ1) was also associated to metastatic risk in breast cancer patients [130]. Higher expression of PLCγ1 and its activated forms in tumour samples, was associated with a higher frequency of distant metastasis, highlighting how PLCγ1 not only could be a potential therapeutic target, but also a prognostic marker for metastatic risk in breast cancer patients [130].

Inhibition of β-oxidation also represents a potential therapeutic for interventions preventing cancer metastasis. Carnitine palmitoyl transferases (CPT) are responsible for the transport of fatty acids to the mitochondria for β-oxidation. In TNBC cells, mitochondrial β-oxidation is upregulated in order to produce sufficient amounts of ATP necessary for survival and proliferation, leading to activation of Src. Inhibition of CPT-1 and -2, by specific inhibitors or genetic knock-down, was able to prevent Src activation, tumour growth and also metastasis formation [131].

The importance of lipids in metastatic spreading was further demonstrated in a recent report exploring lesions generated in the oral cavity of mice after implantation of cells isolated from human oral carcinomas [132]. They found that a subset of CD44^+^ cells was characterized by the expression of genes involved in proliferation, lymphatic metastasis and neoplasm metastasis. Furthermore, these cells also upregulated genes involved in the metabolism and translocation of fatty acids, as well as receptors involved in fatty acid uptake, such as CD36. CD36 was found to be crucial for metastasis formation, enabling cells with low metastatic potential to significantly increase their capacity to colonize lymph nodes upon overexpression. Consistently, knock down of CD36 greatly reduced metastasis formation. Importantly, this effect was only observed in secondary tumours, with little or no effect on the growth of primary lesions, pointing to the specific role of CD36 in metastasis. When mice were fed a HFD (enriched in fatty acids) or cells were treated with saturated fatty acids such as palmitic acid, the number of CD36^+^ cells and metastasis was significantly increased. While both CD36^+^ and CD36^-^ cells were able to form primary tumours, only the CD36^+^ population produced metastasis; administration of two different neutralizing antibodies against CD36 completely inhibited metastasis formation or their size and number when metastasis where already established. Finally, as upregulation of CD36 correlates with poor prognosis and survival rate in patients with different cancers, and that its amplification correlates with metastasis in many human cancers, CD36 appears to be an interesting target for the management of metastatic disease [132]. Several other reports have demonstrated a strong correlation between aberrant lipid metabolism, EMT and metastasis formation [133–136], also highlighting the importance of the research in this field to exploit lipid metabolism as a therapeutic target.

## CONCLUSIONS AND FUTURE PERSPECTIVES

In the context of immunology, two main conclusions can be drawn regarding the importance of fatty acids for immune cells: the generation of immunological memory and the differentiation in specific T cell subsets **(Figure 1)**.

With regard to the generation of T_m_ cells, the majority of reports to date suggest the dependence of these cells on fatty acid β-oxidation and fatty acid biosynthesis mostly as energy source for survival. This finding is of great importance for research questions trying to better understand and manipulate the generation of memory in different pathological contexts. As inhibition of fatty acid biosynthesis is highly detrimental to T_m_ cells, improving or protecting the activity of this pathway through the development of agonists or via administering substrates to boost it and help building an efficient memory compartment, can provide opportunities for the development of therapeutics to combat chronic disease.

Equally important is the variety of fatty acid substrates and their role in dictating the differentiation of T cells, which can be exploited for the generation of clinical interventions aimed at controlling the pathogenicity of aberrant T cells in inflammatory conditions. This may be of particular relevance to autoimmune diseases, where the balance between Th17 and T_reg_ cells is crucial. Activation of PPARγ and inhibition of ACC1 could become a good strategy to block the generation of Th17 cells [45, 50]. This approach can also improve the generation of protective T_reg_ cells [50]. The receptor CD5L has also been found to be a repressor of the pathogenicity of Th17 cells by regulating fatty acid and cholesterol metabolism [53].

Other immune cell types, such as ILC1 and B cells, are also important in the establishment of metabolic disorders, particularly obesity and insulin resistance [62, 63]. Understanding the mechanisms by which these cell types utilize fatty acids and appreciating their exact involvement in maintaining adipose tissue inflammation, typical of obesity, will provide useful insights for studies aiming to combat this condition. Further research in the field of immunology and fatty acid metabolism will provide valuable new information and, more importantly, identify new targets to manipulate the fatty acid metabolic pathways in order to fine balance the generation and function of diverse immunological populations.

Cancer cells also rely upon fatty acids for the production of ATP, which they require in great amounts to meet the energy demand necessary for a high proliferation rate, in the relatively nutrient-poor tumour microenvironment. Inhibiting uptake, biosynthesis and/or utilisation of fatty acids, via targeting the relevant enzymes, receptors and FABP, represents possible strategies to fight cancer **(Figure 2)**.

The inhibition of receptors like CD36 and enzymes involved in fatty acid biosynthesis has shown encouraging results in certain cancers (e.g. prostate cancer- [90]) where fatty acids appear to be important sources of energy to sustain proliferation and survival [91]. The consumption of fatty acids via β-oxidation is also a very important metabolic pathway in cancer cells and that is why some cancers display an entire transcriptional programme aimed at utilizing fatty acids for proliferation [105, 106]. This is the case of the two transcription factors MYC and β-catenin in breast cancer [105] and HCC [106], respectively, both strongly promoting β-oxidation.

Despite their dependence on certain metabolites, such as glucose and diverse types of fatty acids, cancer cells can easily adapt to the fluctuation of nutrients in the tumour microenvironment. The work from Vriens *et al.* [96] shows that cancer cells can use alternative pathways and substrates and that only inhibiting both canonical and alternative signals can be a successful strategy to overcome the metabolic plasticity of cancer cells. Thus, it is evident that along trying to discover new ways of inhibiting canonical pathways, we also need to explore new non-canonical pathways that tumours may exploit for growth and metastasis.

S-acylation is a modification important for the regulation of protein trafficking and function, providing new pharmacological targets that can be exploited in the context of a wide range of pathological conditions, spanning from infection to cancer [75, 114]. Inhibition of this process by limiting the availability of fatty acids or direct pharmacological inhibition of acyltransferases, could prove a viable approach applicable to different clinical scenarios.

Metastatic disease represents one of the greatest challenges of our time, and it is exciting to see an increased number of reports showing that fatty acid uptake and metabolism are actively involved in this process. Inhibition of several enzymes, both of the fatty acid biosynthesis and β-oxidation pathways, can reduce metastasis in many different cancers, while targeting the receptor CD36 represents an efficient strategy for blocking their generation and growth [132].

Future research needs to focus on the most promising targets in order to develop efficient pharmacological tools that can be used in a number of pathological contexts. We also need to further dissect the molecular mechanisms regulating the biosynthesis and catabolism of fatty acids, in order to discover new targets and improve the strategies that would allow us to modulate these processes when they become aberrant.

## AUTHOR CONTRIBUTION

All authors contributed to the manuscript writing, read and approved the submitted version.

## Abbreviatons

ACC: – acetyl-CoA carboxylase,
ACLY: – ATP citrate lyase,
CPT: – carnitine palmitoyl transferases,
CRPC: – castration-resistant prostate cancer,
DHA: – docosahexaenoic acid,
EAE: – experimental autoimmune encephalomyelitis,
EMT: – epithelial to mesenchymal transition,
EPA: – eicosapentaenoic acid,
FABP: – Fatty acid binding protein,
FADS: – fatty acyl-CoA desaturase,
FAS: – fatty acid synthase,
GA: – gastric adenocarcinoma,
HCC: – hepatocellular carcinoma,
HDAC: – histone deacetylase,
HFD: – high fat diet,
ILC: – innate lymphoid cell,
LAL: – lysosomal acid lipase,
LMW-E: – low molecular weight isoform of cyclin E,
LPS: – lipopolysaccharide,
mCRPC: – metastatic CRCP,
NK: – natural killer,
PI(4,5)P2: – phosphatidylinositol (4,5) bisphosphate,
PLD: – phospholipase D,
PPAR: – peroxisome proliferator-activated receptor,
PUFA: – polyunsaturated fatty acid,
SCD: – stearoyl-CoA desaturase,
SREBP: – sterol regulatory element binding protein,
T_conv_: – conventional T cell,
TCR: – T cell receptor,
T_eff_: – T effector cell,
Th: – T helper cell,
T_m_: – T memory cell,
TNBC: – triple-negative breast cancer,
T_reg_: – regulatory T cell,
wt: – wild type.
